# Phenotypic homozygous familial hypercholesterolemia successfully treated with proprotein convertase subtilisin/kexin type 9 inhibitors

**DOI:** 10.1002/ccr3.8537

**Published:** 2024-02-19

**Authors:** Ryosuke Tani, Keiji Matsunaga, Yuta Toda, Tomoko Inoue, Hai Ying Fu, Tetsuo Minamino

**Affiliations:** ^1^ Department of Cardiorenal and Cerebrovascular Medicine, Faculty of Medicine Kagawa University Kagawa Japan

**Keywords:** genetic testing, homozygous familial hypercholesterolemia, pediatric familial hypercholesterolemia, proprotein convertase subtilisin/kexin type 9 inhibitors

## Abstract

Recent data reveal phenotypic HoFH patients may be responsive to PCSK9 inhibitors, challenging prior assumptions. Genetic testing advancements now more accurately forecast patient responses to these therapies, improving treatment strategies.

## Graphical abstract

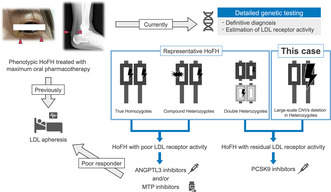

## 1 INTRODUCTION

Familial hypercholesterolemia (FH) is a genetic lipoprotein disorder characterized by elevated plasma low‐density lipoprotein cholesterol (LDL‐C) levels and early onset of atherosclerotic premature coronary artery disease (PCAD).^1^, ^2^, ^3^ This condition manifests distinct clinical features such as xanthomas, thickening of the Achilles tendon, and corneal rings, which often serve as clinical pointers even before genetic confirmation. In particular, homozygous familial hypercholesterolemia (HoFH) is a severe form, presenting with xanthomas and supravalvular aortic stenosis from early childhood and requiring different management from heterozygous familial hypercholesterolemia (HeFH).^4^

In this case, cutaneous xanthomas before the age of 10 years, thickening of the Achilles tendon, and high levels of LDL‐C were suspected to be homozygous HoFH. Historically, our understanding of FH, especially HoFH, suggested that this form was generally unresponsive to proprotein convertase subtilisin/kexin type 9 (PCSK9) inhibitors, primarily because of the absence of low‐density lipoprotein (LDL) receptor activity.^5^ This viewpoint has significantly influenced pharmacotherapeutic decisions, often limiting the available treatment options for patients. This paper reports a case of phenotypic HoFH that was successfully treated with PCSK9 inhibitors.

## 2 CASE REPORT

### 2.1 Case history/Examination

A 29‐year‐old woman presented with cutaneous xanthomas before the age of 10 years and had undergone multiple resections of xanthomas in her teens. The patient was referred to our hospital when xanthomas reappeared. She had Achilles tendon thickening, nodular xanthoma on the elbows, and palpebral xanthoma. No palmar xanthomas or corneal rings were observed.

She had a smoking history of 10 cigarettes per day since the age of 20 years with minimal alcohol consumption. In terms of her family history, her mother suddenly died at the age of 60 years. While the exact cause remains unknown, the circumstances could not rule out the possibility of PCAD. The parents were divorced, and details about the father's family history were unknown. Additionally, her sister had undergone multiple resections for xanthomas during childhood, has been receiving statin therapy, and her LDL‐C levels have been hovering around 140 mg/dL. There was no available information regarding the medical history of other family members.

On examination, her temperature was 36.0°C; her blood pressure was 108/68 mm Hg; her pulse was 60/min; and her oxygen saturation was 98% on ambient air. Blood tests revealed total cholesterol (TC) of 429 mg/dL, LDL‐C level of 357 mg/dL, high‐density lipoprotein cholesterol (HDL‐C) of 45 mg/dL, triglyceride (TG) of 136 mg/dL, and lipoprotein (a) of 20.2 mg/dL, sitosterol of 3.9 μg/mL, lysosomal acid lipase activity of 18.1 μM/h/disk (normal lysosomal acid lipase activity at 14.9 μM/h/disk), thyroid stimulating hormone (TSH) of 1.53 μIU/mL, free thyroxine 3 (FT3) of 2.1 pg/mL, free thyroxine 4 (FT4) of 0.99 ng/dL, total bilirubin of 0.4 mg/dL, aspartate aminotransferase (AST) of 22 U/L, and alanine aminotransferase (ALT) of 30 U/L, hemoglobin A1c (HbA1c) of 5.7% and albumin 4.1 (g/dL). In the urine analysis, there were no signs of proteinuria or hematuria. The abdominal ultrasound did not reveal any findings indicative of hepatobiliary diseases, including primary biliary cholangitis or obstructive jaundice. The Achilles tendon showed a thickening of 15 mm on radiography. Coronary computed tomography angiography was performed, and no coronary artery disease was detected. Additionally, no aortic stenosis was noted.

### 2.2 Differential diagnosis and investigations

In this patient, cutaneous xanthomas before the age of 10 years, thickening of the Achilles tendon, and a high LDL‐C were suggestive of phenotypic HoFH. We also considered other genetic lipid disorders, such as sitosterolemia, lysosomal acid lipase deficiency, and cerebrotendinous xanthomatosis. However, the patient exhibited normal sitosterol and lysosomal acid lipase activities. In addition, the patient showed no neurological symptoms typically associated with cerebrotendinous xanthomatosis.

Following a comprehensive discussion with the patient about the risks, benefits, and implications of genetic testing, informed consent was obtained, and genetic testing was performed as previously reported.^6^ Genetic testing by next‐generation sequencing (NGS) through exome analysis targets 22 genes associated with lipid metabolism abnormalities including low‐density lipoprotein receptor (*LDLR*), apolipoprotein B (*APOB*), proprotein convertase subtilisin/kexin type 9 (*PCSK9*), low‐density lipoprotein receptor adaptor protein1(LDLRAP1), ATP‐binding cassette subfamily G member 5 (*ABCG5*), ATP‐binding cassette subfamily G member 8 (*ABCG8*), apolipoprotein E (*APOE*), and cytochrome P450 family 27 subfamily A member 1 (*CYP27A1*) detected no pathogenic or likely pathogenic variant. In addition, multiple ligation‐dependent probe amplification (MLPA) of *LDLR* revealed a large deletion in exons 7–14 in one allele. This region is crucial for LDL receptor function, and such deletions are categorized as Class 5 mutations, known for causing significant functional defects. The *APOE* genotype was determined to be E3/E3.

### 2.3 Outcome, treatment, and follow‐up

Based on these results, the patient was initially suspected to have phenotypic HoFH, prior to genetic testing. However, subsequent detailed genetic testing revealed it to be genotypic HeFH. Initial therapy with maximum tolerated doses of rosuvastatin and ezetimibe reduced the LDL‐C levels to 152 mg/dL. However, a target LDL‐C level of <55 mg/dL was not achieved. Treatment with a PCSK9 inhibitor (evolocumab) was initiated, considering the residual LDL receptor activity. This intervention proved highly effective, decreasing the patient's LDL‐C level to 22 mg/dL (Table 1). No medication side effects were observed, adequate LDL‐C control was achieved, and the palpebral xanthoma regressed. Written informed consent was obtained from the patient for publication in accordance with the journal's patient consent policy.

**TABLE 1:** Lipid profile of the patient.

Time	TC (mg/dL)	LDL‐C (mg/dL)	HDL‐C (mg/dL)	TG (mg/dL)	Pharmacotherapy
Day 0	429	357	45	136	Pitavastatin 2 mg
					Ezetimibe 10 mg
Day 28	303	246	44	65	Rosuvastatin 10 mg
					Ezetimibe 10 mg
Day 70	220	152	48	99	Rosuvastatin 20 mg
					Ezetimibe 10 mg
					Evolocumab 420 mg q4w
Day 230	91	22	48	103	
Day 400	99	25	56	90	

Abbreviations: HDL‐C, high‐density lipoprotein cholesterol; LDL‐C, low‐density lipoprotein cholesterol; TC, total cholesterol; TG, triglyceride.

## 3 DISCUSSION

FH is a genetic disorder characterized by markedly elevated levels of LDL‐C and significantly accelerated atherosclerotic cardiovascular disease, often resulting in early death. There are two primary classifications of FH: HeFH and HoFH. Individuals with HeFH possess one pathogenic variant and show an abnormal biochemical phenotype. In contrast, those with genotypic HoFH have two variants, which lead to more severe LDL‐C deviations and symptoms. The primary genes associated with these conditions include *LDLR*, *APOB*, *PCSK9*, and *LDLRAP1*. FH, especially the HoFH subtype, is often underdiagnosed, accounting for only approximately 5% of the estimated 30,000 cases worldwide. In recent years, it has become desirable to diagnose the disease in childhood, and genetic testing and universal screening for children has become widespread.^7^ The 2023 European Atherosclerosis Society (EAS) consensus stated that an untreated LDL‐C level greater than 10 mmol/L (approximately 400 mg/dL), along with additional criteria such as the presence of cutaneous or tendon xanthomas before the age of 10 years and/or elevated LDL‐C levels in both parents consistent with heterozygous FH, suggests a likelihood of HoFH. However, genetic testing is typically required to confirm a definitive diagnosis of HoFH. To ensure clarity and avoid misinterpretation, the 2023 EAS consensus recommends using “phenotypic HoFH” as a concise clinical descriptor when a genetic diagnosis is not confirmed, as exemplified in this case report with a large deletion.^8^

**FIGURE 1:**
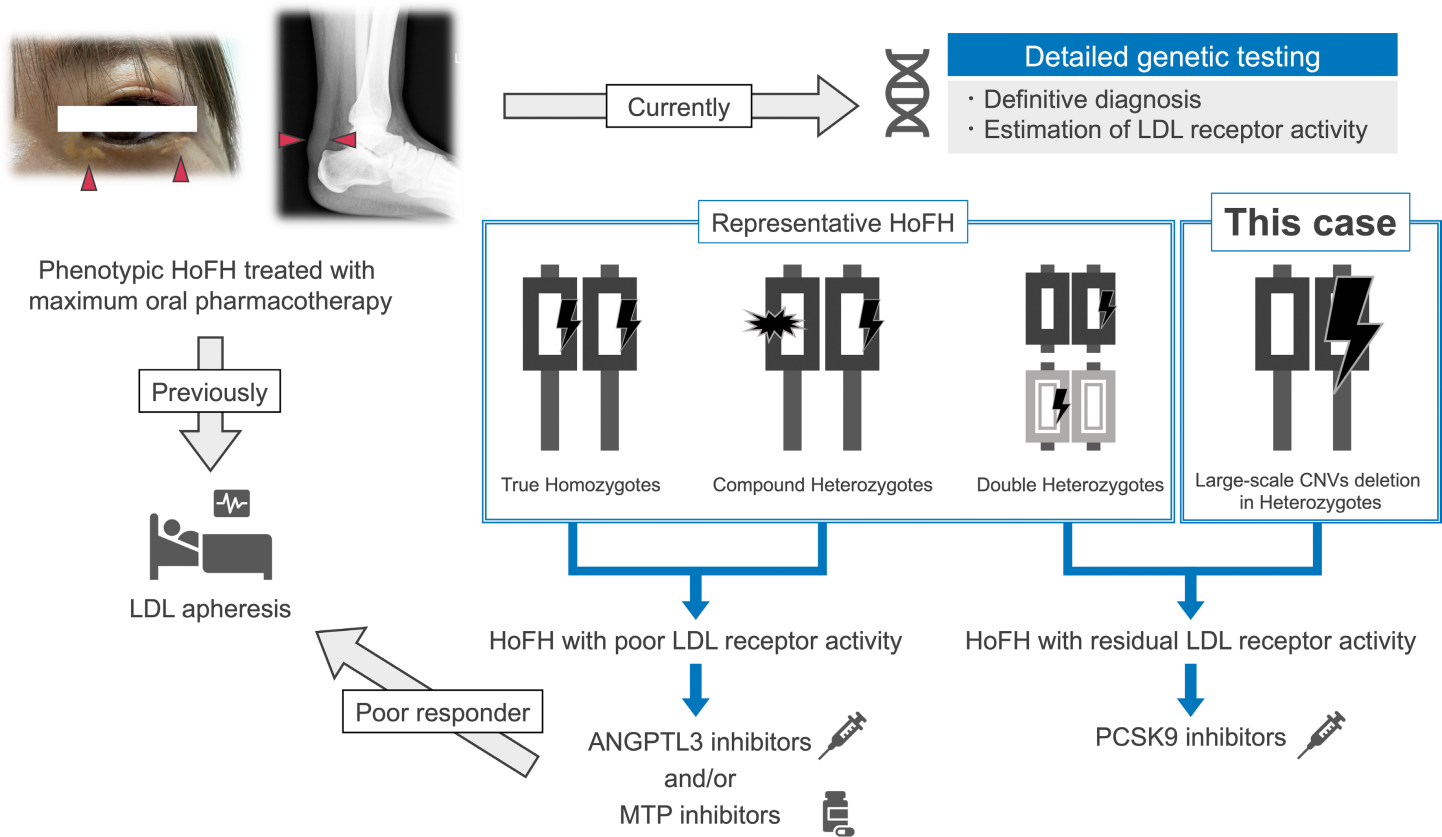
Clinical impact of pharmacotherapy in phenotypic HoFH with large‐scale CNVs deletion in heterozygotes. ANGPTL3, angiopoietin‐like protein 3; CNVs, copy number variations; HoFH, homozygous familial hypercholesterolemia; LDL, low‐density lipoprotein; MTP, microsomal triglyceride transport protein; PCSK9, proprotein convertase subtilisin/kexin type 9.

In this case, despite insufficient information about one parent's family history, the markedly elevated levels of LDL‐C and the presence of cutaneous xanthomas before the age of 10 led to an initial suspicion of phenotypic HoFH before genetic testing. However, following detailed genetic testing, it was revealed to be genotypic HeFH. This case study highlights two important aspects. Some cases of phenotypic HoFH are notably treated with LDL receptor‐mediated pharmacotherapy, including PCSK9 inhibitors. Second, in cases of phenotypic HoFH, detailed genetic testing may help predict the responses to these pharmacotherapies (Figure 1).

The term “phenotypic HoFH” generally encompasses not only true homozygotes but also double heterozygotes and compound heterozygotes.^9^ While rare, genetic variants associated with phenotypic HoFH have been reported to include types resulting from large deletions of genetic material, such as copy number variations (CNVs).^8^

PCSK9 inhibitors are recommended for treating HoFH; however, their effectiveness in reducing LDL‐C varies, achieving an average reduction of 20–35% depending on the type of genetic variants.^10^ Genotypic HoFH in true homozygotes typically exhibits a near‐complete absence of LDL receptor activity; however, there is potential for residual LDL receptor activity in heterozygotes. Therefore, these individuals may benefit from LDL receptor‐mediated pharmacotherapy. In this case, the patient exhibited phenotypic HoFH, though genetic testing later indicated that she was heterozygote with large‐scale CNV deletions and was suspected to have retained some LDL receptor activity. This residual activity likely contributes to the notable effectiveness of the PCSK9 inhibitor.

Further, in cases of phenotypic HoFH, detailed genetic testing may help predict the responses to these pharmacotherapies. The detection rate of pathogenic variants in clinically diagnosed cases of FH is reportedly approximately 60–80%.^11^, ^12^ Genetic testing for FH is not mandatory; it can make the diagnosis of FH more definitive, and if the originator has been genetically tested, the diagnosis of FH in the family is also assured.^13^, ^14^ There are several methods of genetic testing. NGS is renowned for its ability to sequence millions to billions of DNA strands simultaneously.^15^ This allows for a broad examination of substantial genomic regions in a single run. However, implementing this approach is challenging. While NGS can potentially detect variations, such as single nucleotide variants, insertions, and deletions, it is sometimes less reliable for detecting CNVs.

In contrast, MLPA is used to detect large‐scale deletions or amplifications of genes that are difficult to analyze using whole‐exome sequencing methods. The data derived from MLPA are quantitative, rendering an accurate representation of CNVs compared to NGS.^16^ However, they are limited in scope and are incapable of detecting single‐nucleotide variants (SNVs). Most of the pathogenic variants of FH cases are SNVs in *LDLR*, but approximately 10% are attributed to CNVs in *LDLR*.^17^ Therefore, a single genetic test on one side alone may not detect the genetic mutation or may be misjudged. Detailed genetic testing combining multiple genetic tests may provide an efficient algorithm for the genetic testing of FH.^18^ It may be useful in assessing residual LDL receptor activity and predicting the response to these pharmacotherapies in cases exhibiting phenotypic HoFH. In this case, MLPA of *LDLR* revealed a large deletion in exons 7–14 in one allele. Although the LDL receptor activity assay using peripheral lymphocytes has not been measured, these deletions are categorized as Class 5 mutations, known for causing significant functional defects.^19^ It was considered that the diminished LDL receptor recycling capacity observed in Class 5 mutations may have contributed to the response to PCSK9 inhibitors. The point of our case lies in the phenotypic HoFH patient who demonstrated an unexpectedly positive response to PCSK9 inhibitors, emphasizing the need for a deeper dive into individual genetic phenotypes.

### 3.1 Limitation

Some cases of phenotypic HoFH are notably responsive to LDL receptor‐mediated pharmacotherapy, including PCSK9 inhibitors, and detailed genetic testing may help predict the response to these pharmacotherapies. However, further studies are needed to confirm these hypotheses. For cases where PCSK9 inhibitors are ineffective, recent developments in angiopoietin‐like protein 3 (ANGPTL3) inhibitors and microsomal triglyceride transport protein (MTP) inhibitors, which have shown efficacy even in HoFH cases without LDL receptor activity, offer a promising avenue.^20^ These offer the potential to attain the LDL‐C goal or reduce the need for LDL apheresis and show promising potential for determining optimal pharmacotherapies in the future.

## 4 CONCLUSION

While HoFH is traditionally considered ineffective with LDL receptor‐mediated pharmacotherapy, there are phenotypic HoFH cases that demonstrate notable effectiveness with treatments such as PCSK9 inhibitors. Beyond merely confirming a diagnosis, genetic testing also facilitates the prediction of responses to pharmacotherapy, thereby contributing to the advancement of tailored medicine.

## AUTHOR CONTRIBUTIONS

**Ryosuke Tani:** Conceptualization; data curation; investigation; resources; writing – original draft. **Keiji Matsunaga:** Supervision; writing – review and editing. **Yuta Toda:** Data curation; investigation. **Tomoko Inoue:** Data curation; investigation. **Hai Ying Fu:** Data curation; investigation. **Tetsuo Minamino:** Supervision; writing – review and editing.

## FUNDING INFORMATION

None.

## CONFLICT OF INTEREST STATEMENT

None to declare.

## ETHICS STATEMENT

This study was conducted in accordance with the Declaration of Helsinki.

## CONSENT

Written informed consent was obtained from the patient for publication in accordance with the journal's patient consent policy.

## Data Availability

All data generated or analyzed during this study are available as part of the article, and no additional data sources are required.
